# Understanding the Pathophysiology of Preeclampsia: Exploring the Role of Antiphospholipid Antibodies and Future Directions

**DOI:** 10.3390/jcm13092668

**Published:** 2024-05-02

**Authors:** Melinda-Ildiko Mitranovici, Diana Maria Chiorean, Raluca Moraru, Liviu Moraru, Laura Caravia, Andreea Taisia Tiron, Marius Craina, Ovidiu Simion Cotoi

**Affiliations:** 1 Department of Obstetrics and Gynecology, Emergency County Hospital Hunedoara, 14 Victoriei Street, 331057 Hunedoara, Romania; 2 Department of Pathology, County Clinical Hospital of Targu Mures, 540072 Targu Mures, Romania; diana.chiorean@umfst.ro; 3 Department of Pathophysiology, “George Emil Palade” University of Medicine, Pharmacy, Science, and Technology of Targu Mures, 38 Gheorghe Marinescu Street, 540142 Targu Mures, Romania; 4 Faculty of Medicine, “George Emil Palade” University of Medicine, Pharmacy, Sciences and Technology, 540142 Targu Mures, Romania; raluca.moraru@umfst.ro; 5 Department of Anatomy, “George Emil Palade” University of Medicine, Pharmacy, Sciences and Technology, 540142 Targu Mures, Romania; liviu.moraru@umfst.ro; 6 Division of Cellular and Molecular Biology and Histology, Department of Morphological Sciences, “Carol Davila” University of Medicine and Pharmacy, 050474 Bucharest, Romania; laura.caravia@umfcd.ro; 7 Faculty of Medicine, “Carol Davila” University of Medicine and Pharmacy, 050474 Bucharest, Romania; taisia_andreea@yahoo.com; 8 Department of Obstetrics and Gynecology, University of Medicine and Pharmacy “Victor Babes”, 300001 Timisoara, Romania; craina.marius@umft.ro

**Keywords:** preeclampsia, antiphospholipid syndrome, vascular endothelial growth factor, osteopontin, biomarkers, personalized treatment

## Abstract

Preeclampsia (PE) is a hypertensive disorder in pregnancy associated with significant fetal and maternal complications. Antiphospholipid syndrome (APS) is an acquired form of thrombophilia characterized by recurrent venous or arterial thrombosis and obstetric complications that significantly increases morbidity and mortality rates. While preeclampsia may not be the most prevalent obstetric complication in APS, it significantly impacts the long-term health of both mother and child. The treatment of preeclampsia in antiphospholipid syndrome is different from the treatment of preeclampsia as an independent disease. Despite current treatments involving anticoagulants, antiplatelet agents, and antihypertensive drugs, obstetric complications may persist, underscoring the need for cohesive management and effective treatments. The objective of our review is to briefly present knowledge about the physiopathology of preeclampsia and the role of antiphospholipid antibodies in this process. Based on the existing literature, our review aims to identify future directions in molecular pathology toward the discovery of biomarkers and targeted treatments. The application of multidisciplinary approaches and prognostic models, including new biomarkers, could be beneficial in the prediction of PE.

## 1. Introduction

Preeclampsia (PE) is a hypertensive disorder in pregnancy associated with significant fetal and maternal complications such as eclampsia, intrauterine growth restriction (IUGR), intrauterine fetal death, premature birth, and maternal cardiovascular diseases. Globally, it affects 5–7% of pregnant women and ranks as the second leading cause of maternal mortality. PE is characterized by the onset of hypertension and proteinuria after 20 weeks of gestation in previously normotensive women. There are two main types of PE: early-onset PE, which begins before 34 weeks of pregnancy and is less common but more severe, impacting both mother and fetus; and late-onset PE, which occurs after 34 weeks and tends to have milder severity with more favorable neonatal and maternal outcomes. Although the exact etiology of PE remains incompletely understood, it is widely accepted that abnormal placentation plays a crucial role in its development [1,2,3,4,5].

Diagnostic criteria for PE include hypertension (systolic blood pressure of ≥140 mmHg and diastolic blood pressure ≥90 mmHg on two occasions at least four hours apart after 20 weeks of gestation) and proteinuria (≥300 mg per 24 h urine collection or a protein/creatinine ratio of ≥0.3 mg/dL or dipstick reading of 2+). It causes the greatest morbidity and mortality of pregnant women and is responsible for over 70,000 maternal deaths and 500,000 fetal deaths. Additionally, PE may present with thrombocytopenia, renal insufficiency, impaired liver function, pulmonary edema, neurological signs, visual disturbances, or intrauterine growth restriction [2].

Antiphospholipid syndrome (APS) is an autoimmune disorder characterized by recurrent venous or arterial thrombosis and pregnancy morbidity in the presence of antiphospholipid autoantibodies [3,4,5]. APS, an acquired form of thrombophilia, significantly increases morbidity and mortality rates, with obstetric complications such as PE, IUGR, premature birth, miscarriage, and fetal death being well documented. Moreover, thrombotic events are the primary contributors to pregnancy complications in APS, affecting vascular development at the implantation site. APS is estimated to contribute to 6% of all pregnancy morbidity. Clinical features may include hemolytic anemia, persistent thrombocytopenia, nephropathy, cognitive dysfunction, skin ulcers, and catastrophic APS. The estimated prevalence of APS is 50 cases per 100,000, with an annual incidence of 2.1 per 100,000 [3,4,6,7,8,9,10].

Diagnostic criteria for APS include vascular thrombosis and/or pregnancy morbidity. Clinical manifestations encompass vascular thrombosis (arterial, venous, and small vessel thrombosis) and pregnancy morbidities such as recurrent miscarriages, fetal death, preterm delivery, and PE/eclampsia [4,8]. Additional non-criteria clinical manifestations may include persistent thrombocytopenia, hemolytic anemia, livedo reticularis, cardiac valve disease, skin ulcers, nephropathy, cognitive dysfunction, seizure disorders, chorea, and myelitis [6].

Laboratory criteria for APS diagnosis include the presence of lupus anticoagulant in plasma, medium- or high-titer anticardiolipin antibodies (IgG or IgM isoforms), and medium or high anti-beta 2 glycoprotein I antibodies (IgG or IgM isoforms) on two occasions at least 12 weeks apart [4,6,10,11,12,13,14,15].

The treatment of preeclampsia associated with antiphospholipid syndrome is different from the treatment of preeclampsia as an independent disease. In addition to antihypertensive drugs, it includes anticoagulants and antiplatelet agents [4,6,8].

Based on a review of the existing literature, the objective of our study is to briefly present knowledge about the physiopathology of preeclampsia and the role of antiphospholipid antibodies in this process. We aim to identify future directions in molecular pathology to discover biomarkers and targeted treatments. Despite current treatments involving anticoagulants, antiplatelet agents, and antihypertensive drugs, obstetric complications may persist, underscoring the need for cohesive management and effective treatments.

## 2. Materials and Methods

### Protocol and Information Sources

This study adhered to the PRISMA guidelines for conducting reviews [16]. Our search strategy involved the utilization of carefully constructed search strings and was conducted in 2023, focusing on publications from the past decade. Specific keywords, including “preeclampsia”, “antiphospholipid syndrome”, “vascular endothelial growth factor”, “osteopontin”, “biomarkers”, and “personalized treatment”, were employed. A thorough literature review spanning the period from 2015 to 2024 was conducted, utilizing electronic databases such as PubMed and Google Scholar.

**Figure 1:**
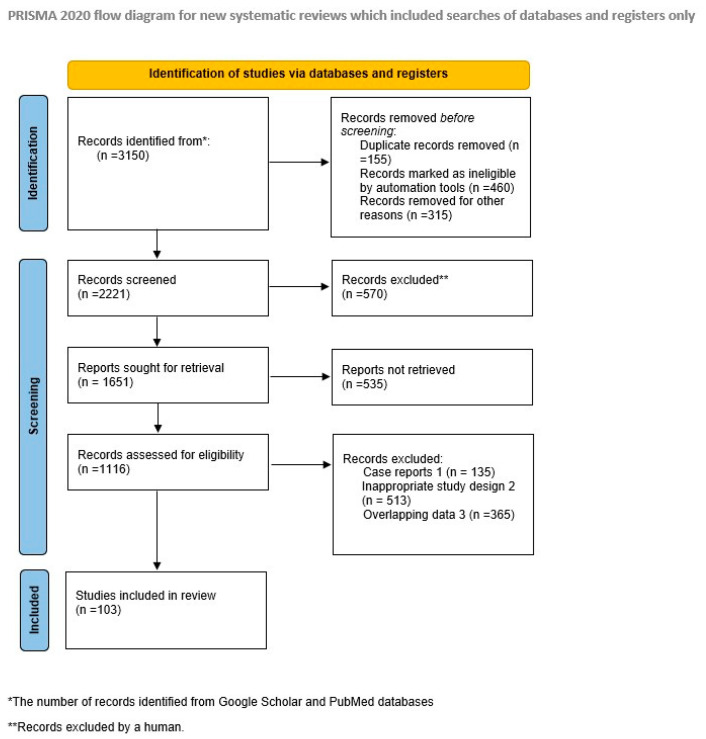
PRISMA 2020 flow diagram for new systematic reviews.

Initially, the literature search yielded a total of 296 titles. The abstracts of articles identified in our search were meticulously scrutinized against predetermined inclusion criteria, which required peer-reviewed, full-text articles written in English and exhibiting appropriate study design, methods clearly defined, with results that were compatible with each outcome domain, without any missing or unclear information. Exclusions comprised books, editorials, literature reports, and studies not aligned with the objectives of this review. Additionally, duplicate findings, case reports, and studies with inappropriate designs were systematically eliminated from consideration. The article selection process involved two authors meticulously assessing the suitability of each study against predefined inclusion and exclusion criteria. Subsequently, pertinent information from the selected studies was independently extracted by the authors. In cases of disagreement, resolution was achieved through consultation with a third author. Ultimately, the selection process yielded a total of 101 articles, which prompted the adoption of a narrative approach given the impracticality of data pooling. A rigorous evaluation of quality, reliability, and validity was conducted for all chosen articles, with any discrepancies promptly addressed. The article selection process is visually depicted in the accompanying flow diagram [Figure 1].

## 3. Physiopathology of Implantation

Due to ethical constraints on studying early placental development, our understanding of placental function remains limited. Instead, we explore the maternal endometrium and the fetal–maternal dialogue [17,18]. Implantation relies on both embryo quality and the physiological state of the endometrium. The ovarian hormones progesterone, estradiol, and relaxin prompt the accumulation of intracellular cAMP (cyclic adenosine monophosphate), synthesized from adenosine triphosphate via the activation of adenylate cyclase. cAMP synergistically enhances decidualization [3,19,20]. The interaction among immune cells, decidual stromal cells, and trophoblastic cells forms the maternal–fetal interface. Any imbalance in this network of cellular connections may result in adverse effects such as preeclampsia. The dynamic changes in immune cells at the maternal–fetal interface have yet to be fully elucidated [21,22].

Implantation of the embryo leads to the invasion of the trophoblast after it adheres to the uterine wall. For this semi-allogenic graft to occur, the endometrium undergoes some modifications. An ”implantation window” opens as a result of decidualization in synchronization with embryonic development. The extravillous cytotrophoblast (EVCTs) cells invade the decidua and then modify the walls of spiral arteries, which are controlled in time and space. Any anomaly in this complex process leads to pregnancy-related disease. To invade the decidua, trophoblastic cells need to recognize, via integrins and cadherins, the components of the membrane and the extracellular matrix (ECM). To control the invasion, the endometrium secretes transforming growth factor (TGF) and tissue inhibitors of metalloproteinases (TIMPs). Moreover, the decidua is colonized by immune system cells, natural killer (NK) cells, lymphocytes, and macrophages, which produce cytokines that have a key role in the invasion of the trophoblast [19,23,24,25]. One crucial role of extravillous cytotrophoblast (EVCTs) cells is to mediate vascular remodeling, which destroys the smooth muscle media. Their dysfunction leads to PE, intrauterine growth retard (IUGR) in normotensive condition, placental abruption, and preterm delivery. Uncontrolled trophoblast invasion in the absence of the decidua results in placenta accreta spectrum [17]. The presence of TIMPs blocks the enzyme activity, thus limiting the invasion in time and space. The most important metalloproteinase (MMP) secreted by trophoblast is MMP9, which is balanced by TIMP1. Moreover, TGF beta is expressed at the fetal–maternal interface and inhibits trophoblast proliferation and invasion [23].

The success of a pregnancy depends on the correct remodeling of the uterine spiral arteries. The invasion of the uterine spiral arteries consists of two successive interdependent phenomena that accomplish the complete transformation of the placenta. The first vascular invasion occurs from 5 to 8 weeks; in this phenomenon, EVCT cells disintegrate the wall of decidual small vessels and create a trophoblastic shell around the vessels, moving from the exterior to the lumen. These intravascular plugs obstruct the decidual capillaries and act as a filter rather than a barrier. This vascular invasion corresponds to the future intervillous space and is followed by a phase of branching angiogenesis. The expression of placental growth factor (PIGF) is moderate during this period, but the expression of vascular endothelial growth factor-A (VEGF-A) and fms-like tyrosine kinase-1 (Flt-1) are immense [21,26,27]. Trophoblastic cells will colonize the surface of the inner wall of the intra-myometrial spiral arteries and then penetrate the vascular tissue. The molecular mechanisms of spiral artery remodeling are still not clear, but the cytotrophoblast cells that enter between maternal endothelial cells lose their epithelial characteristics and acquire an endothelial phenotype [27]. This effect causes the endothelial cells (ECs) and vascular smooth muscle cells (VSMCs) of the tunica media and the internal elastic layer to disappear progressively. The internal elastic layer is then replaced by a fibrin deposit that deprives the vessels of their contractility. The trophoblastic cells progressively develop an endothelial phenotype because of a switch from E-cadherin to VE-cadherin and the acquisition of endothelial cell molecules, such as vascular cell adhesion molecules (VCAMs) [26,28,29]. A low oxygen tension provides a protective environment for the embryo against the teratogenic effects of oxygen free radicals. This environment maintains stem cells in a pluripotent state during the critical period of organogenesis. This is a physiological low-oxygen environment, not hypoxia, with no difference in the adenosine triphosphate-to-adenosine diphosphate (ATP/ADP) ratio; moreover, the placenta is not energetically compromised. The trophoblast plugs begin to disappear at 12 weeks of gestation when hematotrophic nutrition begins and maternal blood is delivered from converted uterine spiral arteries [17]. The second vascular invasion of the intra-myometrial spiral arteries occurs between 13 and 18 weeks [21,26,27]. Much morphological remodeling occurs in the second trimester to optimize the supply of nutrients and oxygen. The formation of terminal mature villi occurs after 20 weeks of gestation and then expands exponentially [17].

The immune system has an essential role in the process of terminal mature villi formation. Decidual natural killer cells (dNk cells) promote embryonic development and are involved in decidual immune tolerance to embryos. Studies have shown that dNK cells participate in trophoblast invasion and spiral artery remodeling [21,23,26,30]. NK cells are implicated in VSMC hypertrophy and disorganization, EC vacuolization, the EVT breaks in the VSMC and EC layers, and dNK secretion of angiogenic factors [26]. Macrophages are categorized into two significant subpopulations: M1, mainly involved in pro-inflammatory responses, and M2, mainly involved in anti-inflammatory responses. M2 is an activated regenerative phenotypical and functional type responsible for immune tolerance and tissue remodeling. Predominantly, the M2 phenotype protects the fetus and placenta.

During the implantation window, decidual macrophages create a pro-inflammatory microenvironment conducive to embryo implantation. Their proximity to spiral arteries induces disruption and disorganization of vascular smooth muscle cells and endothelial cells even before extravillous trophoblasts (EVTs) are present [21]. Inadequate remodeling of spiral arteries due to incomplete trophoblast invasion leads to obstetric complications such as preeclampsia, fetal growth restriction, miscarriage, late spontaneous abortion, preterm birth, and placental abruption [26].

## 4. Physiopathology in Preeclampsia

Early-placentation-impaired trophoblast invasion and defective maternal spiral artery remodeling have been demonstrated in PE. Defective transformation of cytotrophoblast into endothelial cells allows a superficial endovascular invasion [27]. Hypertension and proteinuria are the cornerstones of PE; the disease begins with abnormal placentation with subsequent release of anti-angiogenic factors, which are primarily mediated by fms-like tyrosine kinase-1 and soluble endoglin. Placental ischemia leads to increased angiogenic markers such as soluble fms-like tyrosine (sFlt-1) and soluble endoglin (sEng). Increased levels of sFlt-1 and sEng result in endothelial dysfunction with a negative impact on maternal and fetal organs [2,27,31]. High sFlt-1 levels inhibit podocyte-specific VEGF, disturbing glomerular filtration and contributing to proteinuria. The imbalance of pro-angiogenic and anti-angiogenic factors leads to podocyte injury. Renal biopsy showed diffuse fibrin deposition, endothelial swelling, loss of podocytes, and loss of capillary space. Damaged endothelial cells further induce clotting and loss of anticoagulant ability [2].

Changes in the placenta may also stem from reductions in phosphoinositide 3-kinases genes (PI3K-AKT) and mammalian target of rapamycin (mTOR) signaling. Maternal vascular malperfusion, resulting from the abnormal remodeling of uteroplacental arteries, is acknowledged as a primary precursor to preeclampsia [17]. The incomplete or absent second invasion contributes to diminished blood flow into the intervillous spaces, leading to fetal growth restriction. This condition exacerbates vasoconstriction and platelet aggregation, elevating systemic vascular resistance. Therefore, initiating preventive aspirin treatment as early as 13 weeks, or earlier when obstetric history or pro-coagulant factors are present, is advisable [23].

In early pregnancy, genetic and endothelial cell dysfunction can lead to spasms of the small spiral arteries of the uterus and reduced invasiveness of trophoblastic cells due to ischemia, which ultimately leads to PE. According to Charkievicz et al., the reduced gal-2 may be associated with autoantibodies against this protein and participate in the immunological pathogenic process of PE. Individuals with antiphospholipid syndrome produce antibodies against gal-2. Antiphospholipid syndrome is prevalent in individuals with PE and they have lower levels of galectin-2 (gal-2) [32]. 

Upon exploring additional factors involved in the pathophysiology of preeclampsia, inflammation emerges as a significant player. The inflammasome serves as a molecular link between various components at the syncytiotrophoblast surface and in maternal blood. Consequently, its chronic activation can instigate adverse inflammatory effects, leading to vascular dysfunction and potentially culminating in preeclampsia [33,34]. Moreover, vitamin D deficiency has been linked to pregnancy complications such as preeclampsia and intrauterine growth restriction (IUGR) due to incomplete spiral artery remodeling. Additionally, granulocyte colony-stimulating factor (G-CSF) presents itself as a promising therapeutic target for preventing adverse obstetric outcomes [35]. Furthermore, low preconception complement levels are associated with unfavorable pregnancy outcomes, including adverse pregnancy outcomes (APOs) in antiphospholipid syndrome (APS) and pregnancies among carriers of antiphospholipid antibodies (aPLs) [36]. 

One theory of pathophysiology in preeclamptic headache is that blocking vascular endothelial growth factor (VEGF) and transforming growth factor beta (TGF beta) leads to the loss of fenestrae on the choroid plexus, resulting in endothelial cell instability and periventricular edema. These changes may precipitate seizures, as well as visual disturbances, which may be due to retinopathy, retinal detachment, or cortical blindness. Moreover, cardiac dysfunction increases by 30–50%. The balance between antioxidant capacity and oxidative stress is upset in peripartum cardiomyopathy due to the higher level of sFlt-1. Superoxide dismutase and peroxidase are elevated, and the resulting lack of peroxisome proliferator-activated receptor gamma stat3 upregulates oxidative enzymes, promotes angiogenesis, and mediates cardiomyocyte hypertrophy. Stat3 is decreased in the placenta in cases of PE [2]. Pulmonary edemas occur due to increased vascular permeability, cardiac dysfunction, corticosteroid/tocolytics, and iatrogenic volume overload; the resulting increased vascular permeability damages the endothelial cells [2]. 

Nonetheless, there remains a significant knowledge gap concerning the correlation between antiphospholipid syndrome (APS) and preeclampsia (PE). Antiphospholipid antibodies (aPLs) exert dual effects on the maternal–fetal interface: firstly, they induce a pro-coagulant phenotype by activating various cells such as platelets, monocytes, and endothelial cells, thereby initiating processes such as complement system activation, inflammation, and anti-fibrinolytic effects, ultimately leading to thrombosis. Conversely, aPLs directly impact trophoblastic cells, resulting in apoptosis, impaired proliferation, decreased angiogenesis, and a negative influence on spiral artery remodeling. Preeclampsia manifests as a syndrome affecting multiple organs, characterized by systemic endothelial damage, yet the precise mechanism remains elusive. The dysregulation of angiogenesis appears to contribute to multi-system endothelial dysfunction. While preeclampsia may not be the most prevalent obstetric complication in APS, it significantly impacts the long-term health of both mother and child [37,38], with triple aPLs affecting the obstetric outcome the most [Table 1].

The impact of aPLs on pregnancy outcome is presented below [Table 2].

## 5. Treatment Options in APS

The primary approach in the treatment of APS involves anticoagulation, although some patients still experience thrombosis despite this treatment. Consequently, there is a search for alternative pathophysiological mechanisms and immunomodulating agents. Primary antithrombotic prophylaxis typically relies on antiplatelet agents such as low-dose aspirin for arterial thrombosis [4,50]. On the other hand, secondary antithrombotic prophylaxis usually entails antithrombotic therapy, with vitamin K antagonists being the gold standard treatment for APS. Direct oral anticoagulants like rivaroxaban, apixaban, and dabigatran are utilized for secondary prevention of venous thromboembolism (VTE), although these medications are teratogenic. In pregnant women, accepted treatments include low-molecular-weight heparin (LMWH) and low-dose aspirin [4,50,51,52].

In Christina Han’s study, LMWH improved basal trophoblast migration and induced sFlt-1 increase. The combined therapeutic approach of LMWH and low-dose aspirin promoted migration but did not affect sFlt-1. Antiphospholipid syndrome increased VEGF, PIGF, and sEng and decreased sFlt-1. Antiphospholipid-antibody-induced placental changes were best reversed by LMWH on cytokine, but LMWH worsened the angiogenic changes induced by aPLs with an increase in sFl-1. These findings may explain the inability of current therapies to consistently prevent adverse outcomes [53]. 

Treatment options during pregnancy are restricted to a limited selection of safe medications. However, unresolved issues persist, such as refractory pregnancy loss associated with aPLs and complete heart block linked to anti-Ro antibodies. Among these concerns, perhaps the most significant is the elevated risk of preeclampsia, which occurs three to five times more frequently and complicates 16–30% of pregnancies in women with SLE. Neonatal lupus syndromes, characterized by a range of manifestations including rash, hematologic, and hepatic abnormalities, stem from passively acquired fetal autoimmunity due to maternal antibodies like anti-Ro and anti-La. Although these syndromes typically resolve within six to eight months, cardiac complications may result from permanent damage caused by maternal antibodies [54].

Alternative therapeutic strategies are being explored. For instance, non-steroidal anti-inflammatory drugs, deemed safe during the first and second trimesters, represent one avenue. Hydroxychloroquine, which demonstrates no adverse effects on the baby, has been employed with success. Furthermore, it has been observed that hydroxychloroquine carries a protective effect on endothelial function, although further assessment is warranted [4,54,55,56,57].

Azathioprine stands out as one of the very few immunosuppressive agents with documented safety during pregnancy. However, its dosage should be limited to a maximum of 2 mg/kg/day to mitigate the risk of fetal cytopenias and immune suppression. Tacrolimus and cyclosporine are other immunosuppressive drugs that do not pose an increased risk to the fetus. On the contrary, leflunomide is considered teratogenic. Studies on rituximab and belimumab have shown no significant difference in aPL titers before and after treatments [4,54,58]. Most other agents, such as methotrexate and cyclophosphamide, are contraindicated during pregnancy. However, LMWH and low-dose aspirin are considered safe for use during pregnancy [2,31,34,54,57].

Antithrombotic and anti-ischemic properties of sirolimus and defibrotide are under investigation in APS [4]. Statins, such as pravastatin, which are used for the treatment of hypercholesterolemia and in secondary prevention of atherosclerotic disease, were also used in a study with pregnant individuals with APS who developed PE or IUGR. Pravastatin improved placental blood flow [4,57]. However, other trials, for example, Ahmed et al.’s study (2011-201), did not show any significant reduction in sFlt-1 or improvement in clinical outcomes; furthermore, low compliance was recorded as an adverse effect.

However, the results endorse the safety of pravastatin utilization in high-risk pregnancies. Additionally, limited drug concentrations were observed in the umbilical cord, supporting the notion of restricted transplacental transfer of pravastatin. Lefkou et al. demonstrated that in women with antiphospholipid syndrome and preeclampsia or intrauterine growth restriction (IUGR), the administration of 20 mg of pravastatin led to improved pregnancy outcomes and fetal health [59,60,61]. Furthermore, the efficacy of high-dose intravenous immunoglobulin therapy for pregnant women with aspirin–heparin-resistant secondary antiphospholipid syndrome was also evaluated [62]. Therapeutic options are presented in Table 3.

## 6. Future Directions in Pathophysiology

Limited research has been focused on the role of osteopontin and glycodelin in the pathophysiology of preeclampsia, with even fewer data available for APS. However, evidence suggests that they play significant roles in embryo implantation and development. Evaluating their potential as biomarkers or targeted treatments would be beneficial in future studies. Additionally, investigating their correlation with pro-angiogenic factors like VEGF could provide valuable insights [17].

Uterine secretions contain lipid droplets and glycoproteins such as glycodelin-A and osteopontin, which play important roles in nutritional and immunomodulatory functions at the maternal–fetal interface. Glycodelin-A is implicated in the regulation of extravillous trophoblast invasion and maternal immunotolerance; osteopontin mediates spiral artery remodeling by promoting the migration of smooth muscle cells and it exerts both pro-inflammatory and anti-inflammatory effects. In addition, gland epithelial cells secrete vascular endothelial growth factor (VEGF) and epidermal growth factor (EGF) with angiogenic effects and mitogenic promoters. Perturbed endometrial function may be detrimental to placental development. The use of progesterone improves decidualization and gland function. Moreover, endometrial glands respond to HCG (choriogonadotropin hormone), prolactin, and other hormones by upregulating glycodelin-A and osteopontin. HCG induces the secretion of FGF (fibroblast growth factor), EGF (epidermal growth factor), and VEGF [17,20]. The enhancement of villous fetal capillarization is facilitated by Hofbauer cells, the fetal immune cell type in the villous stroma, which secrete trophic and remodeling factors including osteopontin, VEGF-A, and MMP-9. These angiogenic factors are also produced by secretory-stage decidual NK cells. Alteration in decidual NK cells with a deficiency in osteopontin and osteoglycin can cause complications during pregnancy such as PE and fetal growth restriction [17,21,26]. Diverse genes are implicated in transcriptional expression in VEGF with an impact on placenta development [30]. In the same way, VEGF and osteopontin were found to have an important role in plaque formation in HTA associated with atherosclerosis [67].

In the process of trophoblast adhesion to fibronectin and laminin, additional ligands like osteopontin and fibronectin are involved. These mechanisms are regulated by hormones and other factors such as hCG, prokineticin-1, and leukemia inhibitory factor (LIF), which contribute to receptivity and the maintenance of pregnancy [3,20]. Exploring other factors pertinent to preeclampsia induced by aPLs involves omics research. Various omics, including miRNAs, which are small, single-stranded RNAs approximately 18-24 nucleotides long, are implicated in placental formation. miRNAs play a crucial role in regulating gene transcription, with deregulation of certain miRNAs implicated in PE, particularly in angiogenesis mediated by VEGF [68].

PIGF and sFLT-1 appear to be the biomarkers with the highest sensitivity and specificity in predicting early-onset PE; however, these biomarkers cannot be used to predict late-onset PE or other complications of pregnancy such as IUGR or preterm birth. VEGFs are a family of angiogenic factors whose genetic polymorphism leads to adverse pregnancy outcomes [27]. VEGF-A, PIGF, and the VEGF receptor 1 fms-like tyrosine kinase receptor 1 are expressed in the human placenta and the VEGF family is known as a pro-angiogenic factor implicated in maternal spiral artery remodeling. In addition, a splice variant of FLT-1, designated soluble FLT-1, is expressed in the placenta and is known to have potent anti-angiogenic properties [27]. VEGF, sFlt-1, soluble endogline, antiprothrombin antibodies (aPTs), and PIGF have been evaluated as possible predictors and diagnostic tools. Moreover, uterine artery Doppler is a useful tool [54,69]. Elevated IFN-alfa early in pregnancy could be associated with poor pregnancy outcomes related to angiogenic imbalance in pregnant women with lupus [70].

Osteopontin (OPN) deserves special attention as a biomarker of severe disease as it has been correlated with renal involvement and neuropsychiatric events. OPN levels are significantly higher in cerebrospinal fluid; therefore, OPN could be a novel diagnostic marker. OPN levels are also significantly higher in serum; thus, it could be used as a biomarker for lupus, and OPN and VEGF are found in urine. Clinical manifestations include using OPN as a biomarker for infections, lymphoma, primary aPL syndrome, early rheumatoid arthritis, autoimmune thyroiditis, autoimmune hepatitis, interstitial lung disease, and fibromyalgia [71].

It was also observed that cancers and early pregnancy have similar mechanisms in evolution and development; the major difference is that the process is limited in time and space in the case of pregnancy. However, this fact offers a new approach regarding research models [32].

The result of oncology studies has shown that angiogenesis is regulated in tumors by galectin-1 (GAL-1), and this can be a potential therapeutic target. This pro-angiogenic activity has been demonstrated in early pregnancy by promoting vascular remodeling through VEGF. Moreover, an inhibitor of gal-1 was found to inhibit tumor proliferation, invasion, and angiogenesis. The results of endometriosis studies have confirmed the involvement of gal-1 to be synergic with gal-2 and the role of gal-2 in angiogenesis by activating VEGF. The modulation of gal-2 may become a new therapeutic strategy for stimulating angiogenesis [32].

## 7. Future Directions in Therapy

### 7.1. Nanotechnology as a New Therapeutic Approach

Currently, antihypertensive drugs are the first-line therapy for PE and evidence suggests that low-dose aspirin initiated early in high-risk pregnancies may reduce the risk of development of PE [72]. Extracellular vesicles (EVs) are highlighted as potential novel targets and platforms for therapeutic intervention and/or drug delivery. It is necessary to investigate their biogenesis, biodistribution, metabolism, extraction, and safety and their roles in different organs [73]. EVs contain proteins, nucleic acids, and lipids, and act as messengers for cell-to-cell communication and signaling, particularly between immune cells. Moreover, EVs are known to have roles in reproductive processes [74].

EVs have been investigated in autoimmune diseases, inflammatory disorders, and cancers, but they are also involved in pregnancy complications, particularly in PE. Several studies have shown an increased level of EVs in the maternal circulation in PE; specifically, increased levels of EVs have been associated with PE complicated by fetal growth restriction. Furthermore, EVs may also play a role in the postpartum worsening of the disease. Limited studies have addressed this issue [74,75,76].

Extracellular vesicles are lipid bilayer vesicles released and taken up by diverse types of cells. They serve as facilitators of intercellular communication. In pathological circumstances, they play roles in the aggravation and resilience of various diseases. This is why they hold significant promise in innovative therapeutic approaches for various pathologies. Different nonclinical trials have aimed to provide comprehensive data on the pharmacokinetics and toxicity of EV products. Moreover, EVs have physiological roles in different organs and crucial pathological roles in the development of several diseases [73]. Therefore, EVs are a new powerful therapeutic option for various diseases, providing a potential therapeutic target and, as mentioned, a platform for therapeutic interventions and/or delivery [77].

Furthermore, EVs are currently in the preclinical stage of application and are being investigated for suitable dosage forms for specific applications. Intravenous injection is the most common method of administering extracellular vesicles (EVs). Compared with several nanoparticle (NP) delivery systems, lipid bilayer vesicles offer a versatile platform for drug packing and delivery. Based on their origin, they are classified into synthetically originated lipid particles (LPs), biologically originated EVs, and hybrid liposomes originating from the fusion of LPs and EVs. LPs are self-assembled synthetic nanoparticles that provide a prominent platform consisting of fatty acids and lipids centered in a spherical bilayer membrane surrounding an aqueous chamber. Moreover, LPs have a significant advantage in the context of drug delivery. Additionally, EVs have shown great potential for integrating many small molecules for therapeutic and diagnostic applications [73]. EVs can carry matrix-remodeling enzymes such as metalloproteinases as well as their regulators, contributing to modifications of the extracellular matrix (ECM) [72,78].

EVs originating from dendritic cells, B lymphocytes, and macrophages possess significant therapeutic potential without the need for specific manipulation, exhibiting anti-apoptotic, anti-inflammatory, pro-angiogenic, and anti-proliferative effects. Enhancing the therapeutic potential of EVs can be achieved through engineering EV-producing cells [8]. Additionally, monitoring pEXO expression profiles during pregnancies could offer insights into pregnancy outcomes and serve as novel predictive biomarkers for preeclampsia [74,79,80].

Nanomedicine platforms can be designed to achieve more efficient cytosolic localization, which is a crucial consideration for the delivery of mRNA therapeutics. The cytosol is responsible for protein translation within the cellular machinery [81].

### 7.2. Cancer-Based Therapy

As previously highlighted in this article, there are similarities between cancers and early pregnancy. While conducting diverse research during pregnancy is unethical, studies in cancer are more advanced, holding potential applicability to pregnancy in the future. In cancer treatment, the combination of PD-1 checkpoint blockade has further improved efficacy, resulting in a 35% tumor regression rate. Additionally, mRNA-based vaccines can enhance the effectiveness of CAR T-cell therapy. CARs are genetically engineered receptors that redirect T-cells to identify and eliminate a specific target antigen [81,82].

Manufacturing mRNA delivery vehicles requires the establishment of new approaches for purification, quality control, translational control, long-term stability, and novel stabilizer excipients, leading to widespread use [81]. The monoclonal antibody mAb 1N11 prevents aPL antagonism of endothelial cell migration in mice; however, further studies are needed [82].

### 7.3. Stem Cell Therapy

Mesenchymal stem cells are the most frequently used stem cells in clinical trials due to their easy isolation from various adult tissues, their ability to home to injury sites, and their potential to differentiate into multiple cell types. All the soluble factors and vesicles secreted by MSCs are commonly known as secretomes. MSC secretomes have a key role in cell-to-cell communication and are actively involved in immune modulation and regeneration. The secretome composition has a key advantage in cell-based therapies with a large number of therapeutic possibilities including pregnancy complications [34,72,83].

### 7.4. Angiogenic Factors

Treatment with angiogenic factors was taken into consideration in the case of PE. Studies have demonstrated the role of microvascular injury in HTA and have demonstrated that treatment with an angiogenic factor aimed at ameliorating microvascular and renal injury would prevent the development of HTA. Cyclosporine was administered in rats with renal lesions with afferent arteriopathy. The rats received vascular endothelial growth factor as treatment, which resulted in lower blood pressure. VEGF treatment was also associated with a decrease in osteopontin expression. Moreover, treatment with VEGF reduces the hypertensive response and accelerates histological recovery. VEGF also has a vascular-protective effect [84].

It has been reported that circulating fms-like tyrosine kinase 1 sFlt-1 is an anti-angiogenic factor while placental growth factor PIGF is a pro-angiogenic factor; therefore, the sFlt-1/PIGF ratio demonstrated the highest significance in PE. VEGF is a key regulator of vascular function and angiogenesis. Further, soluble endoglin, cell-free fetal DNA, and vasoactive factors have been highlighted as prospective predictive biomarkers for PE [79]. The administration of VEGF in rats with chronic nephropathy and HTA improved their condition [84]. Moreover, the effect of VEGF on osteopontin expression in the renal cortex in microscopy showed a significant decrease of 1.4 ± 0.5 vs. 2.3 ± 0.2% [84]. The most plausible effect of VEGF is the mechanism by which VEGF lowers blood pressure by accelerating recovery from renal tubulointerstitial and microvascular injury. It dramatically improved arteriopathy [84].

Furthermore, VEGF treatment also results in lower blood pressure in pregnancy. A previously described model of preeclampsia was reproduced by adenoviral overexpression of the soluble vascular endothelial growth factor VEGF receptor sFlt-1 in pregnant and nonpregnant rats. The animals were treated with recombined VEGF 121 at 0, 100, 200, and 400 micrograms/kg once or twice daily and compared with normal rats [85].

VEGF121 treatment alleviated the symptoms and reversed the sFlt-1 changes in gene expression, demonstrating that VEGF121 had beneficial effects on PE. VEGF is a platelet-derived growth factor that induces vascular health by suppressing endothelial apoptosis, inhibiting leukocyte adhesion, and inhibiting platelet aggregation and thrombosis. The biological role of VEGF depends on its interaction with two receptors, VEGFR1 and VEGFR2. Deprivation of VEGF activity is induced by overexpression of sFlt-1 or by VEGF antibodies. Therefore, agents that bind sFlt-1, such as VEGF121, may be a useful targeted therapy in PE [85].

These agents reduce endothelial swelling and fibrin deposits, provide a good histological score of *p* < 0.05, and are dose-dependent. A significant reduction in blood pressure with *p* < 0.01 was recorded, along with a reduction in urinary albumin per creatinine. The effects on gene expression were shown to reverse genes. The expression levels of six genes encoding soluble secreted proteins were affected by sFlt-1 transfection, PAI-I, MMP-9, MMP-12, osteopontin, and IGFBP5 [85].

## 8. Discussion

APS is a thrombo-inflammatory disease with a variety of clinical phenotypes. Thrombosis prophylaxis should take an individualized risk stratification approach. Obstetric management is focused on close monitoring of the fetus and mother and low-dose aspirin in asymptomatic aPL carriers. In patients with a history of PE or fetal death, treatment with low-dose aspirin combined with LMWH is recommended; in women with a history of thrombosis, treatment with low-dose aspirin combined with LMWH is recommended; in women requiring postpartum management with no history of thrombosis, treatment with LMWH for 6 weeks is recommended; and in women with a history of thrombosis, treatment with warfarin or LMWH is recommended [6,10,11]. In addition, other pharmacological agents should be considered, such as hydroxychloroquine and statins. Moreover, the implication of neutrophil extracellular traps in thrombin generation and initiation of inflammatory cascades is a relatively recent discovery, but more data are needed [6,10,11,12].

Regarding medication safety, non-steroidal anti-inflammatory drugs are generally accepted during pregnancy, except in the third trimester, which may cause premature closure of the ductus arteriosus [86]. Other studies showed that NSAIDs are associated with miscarriage, low birth weight, and premature closure of the ductus arteriosus [87]. Glucocorticoids and prednisone can lead to preterm birth, low birth, FGR, and malformations of oral clefts [86,87]. Low-dose aspirin and LMWH are safe for the prevention of preeclampsia. Antihypertensive medications are widely used such as methyldopa and hydralazine, but angiotensin II receptor blockers are contraindicated during pregnancy. Hydroxychloroquine is encouraged because of its safety profile [87].

However, as indicated by other studies, hydroxychloroquine can lead to preterm delivery and low birth weight depending on the dosage [86,88]. Among immunosuppressive agents, cyclophosphamide, methotrexate, and mycophenolate are contraindicated in pregnancy due to their association with congenital malformations, impacting the heart, central nervous system, and skeleton [86,87]. Direct oral anticoagulants (DOACs) should be avoided [89]. Azathioprine is considered relatively safe. Cyclosporine also seems to be an acceptable option. Anti-TNF and rituximab do not seem to place the mother or fetus at risk [86]. However, according to other studies, tumor necrosis factor inhibitors (anti-TNF therapy) have been linked with VACTERL defects. Rituximab can be implicated in premature birth and hematologic abnormalities, but we have limited data [87,89]. Sulfasalazine in some studies has shown a risk of preterm birth, low birth weight, and abnormalities; leflunomide is similar to methotrexate; and tocilizumab is not related to significant problems [86]. Moreover, plasma exchange is a safe and efficient option [90]. Potential new therapies such as Coenzyme Q improve endothelial function; adenosine receptor agonists suppress antiphospholipid antibody-mediated NETs (neutrophil extracellular traps) release, or agents targeting antibody-producing cells, but further studies are needed [89].

Despite the use of antiplatelets and anticoagulants, women with APS develop pregnancy complications [91]. As shown by Liping Liu in a review of eight different studies with a total number of 212,954 participants, APS has been associated with significantly worse pregnancy outcomes [92]. One-quarter of maternal deaths are due to PE. Therefore, PE screening is essential to prevent complications. Preeclampsia screening is based on maternal risk factors and has a slow detection rate. The multiple theories behind PE etiology should be the scientific base for establishing the risk factors [93,94].

Risk factors already established are previous pregnancy with PE, multiple gestation, history of small-for-gestational-age delivery or adverse outcome, age extremes, lack of seminal exposure, immunologic mismatch and absence of relaxin, pre-existing patient health conditions (thrombophilia, autoimmune disease such as APS and SLE, obesity, chronic hypertension, and diabetes mellitus), gene predisposition, and sociodemographic condition [93,95,96,97].

Regarding biomarkers for screening, there is a lack of consensus among clinicians and researchers: in 2018, the International Society for the Study of Hypertension in Pregnancy recommended against the use of PIGF. Furthermore, ACOGs are not convinced of the predictability of the statistical models because the heterogeneity of the predictors’ external validation is difficult to perform [93].

Low-cost technologies are sought in the prevention and treatment of obstetric complications. Preconception analysis is an unmet need and there is a lack of biomarker specificity. Moreover, there are discrepancies between studies due to differences in trophoblast preparation methods, and differences are found in the quantification of circulating biomarkers due to different analysis methods. Machine learning could be useful. In addition, alternative biological samples, such as follicular fluid, should be considered for performing PE biomarker studies. We can also improve the prediction of adverse pregnancy outcomes in women with SLE and APS using machine learning [93,98,99].

The Fetal Medicine Foundation (FMF) suggested a screening paradigm based on uterine artery pulsatility index and biochemical indicators, PIGF, and the pregnancy-associated plasma protein A (PAPP-A) which predicts preterm PE at a 75% rate. At present, some biomarkers such as VEGF, sEng, sFlt-1, placental protein-13 (PP-13), growth differentiation factor 15 (GDF15), a disintegrin and metalloprotease 12 (ADAM12), and inhibin alpha are considered as predictive factors for risk classification for PE pregnancies, but no clinical relevance has been found so far, even if there are studies that would show that, for example, the sFlt-1/PIGF ratio seems to have a high predictive value [100,101].

There is a need for strategies to improve pregnancy outcomes in patients with SLE and APS, such as appropriate preconception, strict disease control before pregnancy, medication adjustment, and intensive surveillance during and after pregnancy by obstetricians and rheumatologists [87,102,103].

## 9. Conclusions

In conclusion, a detailed history of symptoms suggesting a systemic connective tissue disease can be useful in the prevention of obstetric complications. Family planning should be discussed as early as possible after diagnosis. A pregnancy could be successful if measures are taken to reduce the risk of obstetric complications. Furthermore, pregnancy can affect autoimmune disease progression. Risk stratification includes disease activity, autoantibody profile, previous vascular thrombosis, hypertension, the use of drugs such as anticoagulants/antiplatelets, and HCQ. Future directions in therapy are necessary. Therefore, the application of multidisciplinary approaches and prognostic models, including new biomarkers, could be beneficial in the prediction of PE.

## Figures and Tables

**Table 1:** Obstetric outcomes.

Studies	Cohort	aPL Positivity							Other Antibody Positivity
Karen Gibbins 2018 [39]	148 women with preterm delivery PREPI	11.5%							
	148 women with preterm delivery controls	1.4%							
Ryan Malcolm Hum 2022 [40]	98 patients with no controls	26%							32% Ro autoantibodies
Maria-Grazia Lazzaron 2019 [41]	Adverse pregnancy outcome	aPL single positive 2 (5%)	aPL double positive 0	aPL triple positive 4 (44.4%)					
	Thrombosis	1 (2.5%)	0	1 (11.1%)					

aPL-positive antibodies are strongly associated with PREPI. Triple aPL-positive antibodies are related to a higher risk of adverse pregnancy outcomes.

**Table 2:** The impact of aPLs on pregnancy outcome.

Studies	Study Cohort	Miscarriage	Fetal Death (Stillbirth)	Neonatal Death	Preterm Delivery (PREPI)	Preeclampsia	Small for Gestational Age
Jill P Bujon 2015 [42]	385 patients with LAC/0 controls	0	4%	1%	9%	9%	10%
Ryan Malcolm Hum 2022 [40]	98 patients (31 with aRo, 25 with aPL)/0 controls	2%	1%	0	0	1%	0
Maddalene Larosa 2022 [43]	238 LAC/0 controls	3.4%	2.9%	0.4%	2.9%	2.1%	2.1%
April Barnado 2014 [44]	577 aPL or lupus	124 (21.49%)	16 (2.77%)	0	66 (11.43%)	45 (7.8%)	58 (10.05%)
	694 controls	85 (12.24%)	5 (0.7%)	0	29 (4.18%)	29 (4.18%)	28 (4.03%)
Jing Liu 2022 [45]	aPL with HCQ 93	66.1%	5.1%	0	0	13.6%	6.7%
	aPL without HCQ 46	43.2%	2.7%	0	0	16.2%	5.4%
Mohamed Ibrahem Eid 2019 [46]	aPL-positive, LMWH at positive pregnancy test 48	27.08%	0	0	10.41%	16.66%	14.53%
	aPL-positive, LMWH at cardiac activity confirmation 46	43.47%	0	0	8.69%	15.21%	10.86%
Elefteria Lefkou 2016 [47]	aPL with PE during LDA plus LMWH control 10	Not applicable	50%	0	100%	100%	No data
	aPL with PE during LDA plus LMWH, cohort with pravastatin 11	Not applicable	0	0	0	100%	No data
Shanying Chen 2015 [48]	LAC pregnancies after 6-month remission 52	17.3%	3.84%	0	7.69%	9.61%	
	LAC with active lupus during pregnancy 13	61.53%	15.38%	0	15.38%	7.69%	
	LAC discovered during pregnancy 19	42.10%	10.52%	0	26.31%	5.26%	
Zeynep Belce Erton 2021 [49]	55 patients aPL-positive/no controls	27%	12.5%	0	22.5%	5.45%	0

LAC—lupus anticoagulant-positive; aPL—antiphospholipid antibodies; HCQ—hydroxychloroquine; LDA—low-dose aspirin; LMWH—low-molecular-weight heparin. aPL—associated with a high risk of adverse pregnancy outcomes. HCQ only ameliorated miscarriages; moreover, the administration of LMWH from the positive test of pregnancy has no significance and is only related to miscarriage. If pregnancy is planned, a favorable outcome could be achieved.

**Table 3:** Therapeutic options.

Studies	Cohort	LMWH	HCQ	HCQ + LDA	Prednisone	LDA + LMWH	Immunosuppressive Drugs/Tacrolimus	LDA	Other Drugs/ No Drugs
Maddalena Larossa (2022) [43]	238 women with LAC/no control (remission in 86.6%)	0	98.3%	0	50%	0	23.5%	69.3%	0
Rubine Izhar (2021) [63]	aPLs during pregnancy PREPI 98	21.4%	0	0	0	0	0	41.8%	0
	aPLs during pregnancy without PREPI 106	2.9%	0	0	0	0	0	7.5%	0
Shangqin Long (2023) [64]	aPL-positive with preeclampsia	0	0	0	0	36.36%	0	31.58%	65% no medication
	aPL-positive with fetal loss	0	0	0	0	0	0	10.53%	35% no medication
Na Zhang (2022) [65]	90 patients with LAC, adverse outcomes: HTA		17.8%	2.2%					
	PE		11.1%	0					
	Preterm delivery		33.3%	17.8%					
	Small for gestational age		18.9%	9.5%					
	Neonatal asphyxia		16.2%	2.4%					
Takehiro Nukai (2024) [58]	LAC-positive patients with Maternal APO						44.8% (from a total of 29)		Other medication: 26.2% (from a total of 95)
	Neonatal APO						51.7%		44.2%
	Preeclampsia						10.3%		5.3%
	Small for gestational age						34.8%		35.2%
	Major malformations						4.3%		1.1%
	Neonatal death						4.3%		0
	Maternal death						0		
Rahana Abd Rahman (2020) [66]	Patients with LAC	HCQ							non-HCQ */other medications
	Outcomes: HTA	6.4% from 47							42.9% from 35
	Preterm delivery	44.68%							60%
	PE	11.8%							33.3%

HCQ—hydroxychloroquine; LDA—low-dose aspirin; LMW—low-molecular-weight heparin. * HCQ improved the prevention of PE; moreover, LDA + LMWH showed efficacy, LAC-lupus anticoagulant, APO-adverse phospholipid outcome, PE-preeclampsia, aPL-antiphospholipid antibodies.
